# Differential Analysis of the Years of Experience of Higher Education Teachers, their Digital Competence and use of Digital Resources: Comparative Research Methods

**DOI:** 10.1007/s10758-021-09531-4

**Published:** 2021-06-25

**Authors:** Francisco. D. Guillén-Gámez, Julio Cabero-Almenara, Carmen Llorente-Cejudo, Antonio Palacios-Rodríguez

**Affiliations:** 1 Faculty of Education Department of Didactitics and Scholar Organisation, University of Cordoba, Cordoba, Spain; 2 Faculty of Education Sciences Department of Didactics and Educational Organization, University of Seville, 41013 Seville, Spain

**Keywords:** ICT resources, Digital competence, Teachers, Educational experience, Higher education, Educational research methods

## Abstract

This study compares the level of digital competence of Spanish higher education teachers in the use of three types of ICT resources in the classroom: digital tools to consume information, digital tools to produce information, and emerging technologies. To measure the level of competence, the validated DigCompEdu Check-In instrument with an ex post facto design is used. The sample consists of 2180 university teachers from Andalusia (Spain) working in different areas of knowledge: Arts and Humanities, Sciences, Health Sciences, Engineering and Architecture, Social and Legal Sciences; and classified into three levels of teaching experience: between 0–5 years, 6–14 years, and 15 years or more. The results show that teachers with 15 or more years of experience represent the group with the most significant differences in regard to their level of digital competence when comparing the use of the three types of ICT resources; furthermore, the results were similar for all areas of knowledge. In addition, the visualization or creation of videos, as well as the visualization or creation of posters and concept maps, are the resources that were found to be most significant for the three levels of experience. For each specific area, it is recommended that each of their results is analysed in detail. Finally, further research is recommended to validate these preliminary findings in each of the knowledge areas.

## Introduction

The emergence of Information and Communication Technologies (ICT), especially with the arrival of the Internet in a way accessible to the general public, focus all efforts on the development of Learning Management Systems (LMS), collaborative tools for editing and sharing information or communication and interaction tools, among others, which represent the key to the effective use of these ICT resources by teachers for powerful ongoing training (Liesa-Orús et al., 2020).

However, since the integration of ICT into educational contexts, a number of problems have become evident, since the focus of studies and research has been on technological tools and their technical potential, and not on the training required to incorporate them didactically into teaching and learning processes (Specifically in the field of higher education) (Cabero-Almenara & Llorente-Cejudo, 2005; Cabero-Almenara, 2007; Jackson, 2017; Finnegan & Ginty, 2019; Padilla-Hernández et al., 2020). Previous studies have found some deficiencies in the level of technological and pedagogical training, both among teachers and students (Silva et al., 2019; Guillén-Gámez et al., 2020b), and have suggested that these competences could be improved by continuously being in contact with ICTs. The global COVID-19 pandemic has highlighted, through countless media and social networks, needs that are barely being met and others that have not been adequately addressed (Auma & Achieng, 2020; Daniel, 2020; Murphy, 2020; Ortiz, 2020).

Taking into account the complex, dynamic, and uncertain educational panorama, professionals in the field of education recognise that teachers and students’ future professional skills will require the incorporation of ICT. Both groups are, on the one hand, subject to a high level of training and motivation regarding the efficient use of these tools (Siddiq et al., 2016; Tondeur et al., 2018); on the other hand, time represents a key component to be able to plan and integrate them effectively (Cabero-Almenara et al., 2020; Drijvers et al., 2010; Prieto-Rodriguez, 2016). For this reason, the need to obtain adequate digital competence training in terms of both time and form is emphasised, allowing course contents to be developed sufficiently and with pedagogical criteria appropriate for students who favour technological resources.

At the same time, there is much emphasis on the study of teachers’ digital competence as a transcendental element of the construction of pedagogical knowledge useful for practice and, consequently, to improving student learning. Digital competence (DC) can be understood as the knowledge, skills, and attitudes that are required to use ICT (Cabero-Almenara & Palacios-Rodríguez, 2020; Calvani et al., 2008). Currently, the digital competence model has evolved to incorporate other specific models for different groups, such as teachers (Ghomi and Redecker, 2018; Redecker & Punie, 2017). There are numerous studies on the development of DC, both for teachers and students (Cabero-Almenara et al., 2017; Cabezas et al., 2020; Guillén-Gámez et al., 2020a), which range in focus from how DC is related to the skills, attitudes, and knowledge required by teachers in a digitalised world, to how to study it from a didactic-pedagogical perspective in a professional educational context.

There are many studies on the creation of instruments or models that allow us to establish measures of the level of technological competence, for example, the self-perception instrument used by González-Martínez et al. (2018), known by the initials INCOTIC, where the use of ICT is measured; this instrument has already been used by other researchers, such as Miravete (2018). In a similar context, the National Institute of Educational Technologies and Teacher Training (INTEF, 2017) developed a rubric based on dimensions, indicators, and levels of skill development in digital teaching competence. Alternatively, Ruiz et al. (2015), focused on evaluating the self-perception of attitudes towards ICT, and technological and didactic knowledge, through the ACUTIC instrument, which has since also been used by other researchers (Guillén-Gámez & Peña, 2020; Guillén-Gámez & Mayorga-Fernández, 2020a). Another model that establishes measures of digital competences is the so-called TPACK model (Technological Pedagogy and Content Knowledge), which has been used in studies and research (Cejas et al., 2016; Cabero-Almenara, 2014; Leiva Núñez et al., 2018) and offers a frame of reference that allows a more meaningful understanding of the different dimensions that comprise digital competence.

## Theoretical Framework

The integration of ICTs in training contexts supposes two fundamental types of digital competence: disciplinary and technological (Cabero-Almenara, 2014; Romero-Tena et al., 2020). The former concerns the development of the skills of the discipline or material; the latter concerns the development of specific digital competences, referring to the management and critical use of integrated technologies in the training context, focused on the objectives of the Horizon 2019 report (Alexander et al., 2019).

Among the most basic resources, the use of videos has been analyzed by some authors. García-Esteban (2017) analyzed the effect of the use of videos as virtual objects on the digital competence of 64 university students of Primary Education through a control and experimental group. The results determined that the students of the experimental group acquired greater competence because the use of virtual videos created an increase in the search, administration and use of digital data. The use of videos as a didactic resource in university education has also been studied by other authors, finding a positive relationship (Cacheiro et al., 2019; Guillén-Gámez et al., 2018; Santos Espino et al., 2020).

Regarding the effectiveness of virtual presentations (PowerPoint, prezzi, genially…), Maharaj-Sharma and Sharma (2017) explored the opinion of teachers and students on the effectiveness of these digital resources. The results found that the teachers had a good opinion about these resources when using them daily with their students. In the same context, in another study by the author Maharaj-Sharma et al. (2017) showed in a sample of 30 science teachers that the use of virtual presentations was the most used resource in teaching. However, other authors have stated that this digital resource offers the same benefits as presentations that are made in a conventional-traditional way (Cosgun Ögeyik, 2017).

If we focus on more emerging resources, authors like Castro et al. (2018) or Miller and Nourbakhsh (2016) consider that educational robotics is an important tool to promote computational thinking and digital competence. However, Esteve-Mon et al. (2019) affirm that at a general level there is still a lack of competences in teachers on how to prepare their students in the didactic use of these resources. More specifically, Román-Graván et al. (2020) found in an experience with this technological resource with future teachers in the area of Social Sciences, that there were no significant differences in the perceptions by age ranges or the academic degree that the students were studying.

Another resource that would provide more stimulating, collaborative and interactive large scenarios is augmented reality (Vázquez-Cano et al., 2020). Regarding this type of resource, López Belmonte et al. (2020) analyzed the perceptions of 428 teachers on the cooperative use of this digital resource, finding that only a minority of teachers implement augmented reality in their classes. Furthermore, there are statistically significant differences in terms of professional experience, which is why younger teachers tend to implement methodologies based on the use of emerging mobile technologies such as augmented reality. These results on the years of experience and the use of this resource were also corroborated by Oleksiuk and Oleksiuk (2020).

Another resource with more projection in the coming decades is virtual reality (Radianti et al., 2020), facilitating the interaction of students from different locations and placing them together in a virtual location in another place (Hernandez-Pozas & Carreon-Flores, 2019), improving knowledge compared to traditional learning (Kyaw et al., 2019), requiring great preparation by teachers (Gómez-García et al., 2018).

In addition to the benefits that these digital resources bring to the teaching–learning process, it is already observed in some of these investigations how age or years of experience affects the development of digital competence (Farjon et al., 2019; López Belmonte et al., 2020; Oleksiuk & Oleksiuk, 2020); although other studies corroborated the opposite (Román-Graván et al., 2020). Therefore, the direction of some general results is not entirely clear about how it affects these variables, requiring further study.

Taking into account the types of studies carried out so far in the scientific literature, the main contribution that the authors make with this study is not only to analyze the level of digital competence of teachers according to the DigCompEdu framework, but also to compare if there are differences significant at this level of digital competence among teachers who use three types of digital resources (information consumers, information prosumer and emerging) against those teachers who do not use them, for each area of knowledge (Arts and Humanities, Science, Health Sciences, Engineering and Architecture, Social and Legal Sciences) and differentiating three ranges of years of experience (0–5 years, 6–14 years, more than 15 years). We believe that with this type of research we take a further step in the study of digital competence from a different perspective than those carried out in related studies, with a triangulation of variables in the study of teaching digital competence: types of digital resources, areas of knowledge of the teaching staff ranges of teaching experience.

With this prior justification, our research question is: Are there differences in the level of digital competence of teachers depending on the age range of experience they have, in relation to whether or not they use different digital resources in the classroom? To answer it, the purpose of this study was:

- To know the level of digital competence of Higher Education teachers in each area of knowledge, depending on whether or not they use digital resources as “information consumers”, as “information prosumer” and “emerging tools”, in three ranges of teaching experience (0–5 years, 6–14 years, more than 15 years).
- To know if there are significant differences in the level of digital competence of teachers in each area of knowledge, between those teachers who do use these digital resources in the classroom compared to those who do not use these resources.

## Method

### Design and Samples

A quantitative methodology was adopted for this research; specifically, an ad-hoc questionnaire was used. The population of this study is 17,321 teachers from higher education in Andalusia (Spain), according to data and figures from the Spanish university system maintained by the Ministry of Science, Innovation and Universities (MCIU, 2019). The sample was purposively selected through non-probability sampling, achieving a response rate of 13.10% (2,262 participants). From the clean-up of the database regarding atypical or lost samples, 2,180 questionnaires were successfully completed. The survey was completed anonymously, preserving the confidentiality of the data.

The teaching staff were assigned to five areas of knowledge: Arts and Humanities, Sciences, Health Sciences, Engineering and Architecture, Social and Legal Sciences. Table 1 describes the number of teachers in each area and their levels of teaching experience.

**Table 1:** Description of the participants over the years of teaching experience

	0–5 years		6–14 years		+15 years		N total
	Female	Male	Female	Male	Female	Male	
Arts and humanities	36	24	34	32	101	111	338
Science	24	12	20	28	83	168	335
Health sciences	51	16	32	36	69	89	293
Engineering and architecture	12	29	30	60	62	226	419
Social and legal sciences	73	28	119	94	233	248	795

### Instrument

The survey is divided into three parts: demographic information is obtained through part A of the survey, with variables on the gender of the teaching staff, the years of teaching experience in Higher Education, age or area of knowledge in which it is developed his teaching and research career.

Part B is DigCompEdu Check-In instrument, which was developed by Ghomi and Redecker (2018) and adapted to the Spanish version by Cabero-Almenara and Palacios-Rodríguez (2020). This measures the level of self-perception in digital competence of Higher Education teachers, with a total of 22 items classified in 6 dimensions. The dimension 1- Professional Commitment focuses not only on using ICT to improve teaching, but also to communicate with the rest of their coworkers. The dimension 2- Digital Resources is focused on the identification, creation and distribution of digital resources. The dimension 3- Digital Pedagogy is related to being able to design, plan and carry out the use of ICT resources, advocating for a change in active and innovative methodologies. Dimension 4- Evaluation and Feedback focused on how teachers use ICT to improve evaluation systems. The dimension 5- Empowering Students is focused on how teachers promote the active participation of students through digital technologies, adapting the level of competence of each student to their interests and educational needs. And, finally, the dimension 6- Facilitate the Digital Competence of the Students, on how to develop and facilitate the digital citizen competence of the students.

To measure the level of self-perception about digital competence, the instrument classifies five progressive levels of management through a five-point Likert scale (from 0 to 4), where each value on the scale gradually alludes to a level of digital competence: A1 (newcomer), A2 (explorer), B1 (integrator), B2 (expert), C1 (leader) and C2 (pioneer), referring to the Common European Framework of Reference for Languages (CEFR).

Regarding the psychometric properties of the instrument, the authors of the instrument had only verified the reliability and validity of the content, so the rest of the tests will be analyzed in this study: exploratory factor analysis (EFA) and confirmatory factor analysis (CFA).

In part C of the questionnaire, questions are asked about the digital tools and resources that the teacher uses for the learning of his students. These resources are classified according to their typology:

- Digital tools used in the classroom for student learning as an information consumer (watch videos, watch slide shows, complete online tests, and view digital posters and concept maps).
- Digital tools used in the classroom for student learning as an information prosumer (create videos, create web pages, create digital posters and concept maps, create online tests).
- Emerging digital tools: disruptive technologies that are consolidating in the educational landscape. It includes the use of robots as a common thread to enhance the development of skills and competencies (robotics); integration of game mechanics into teaching–learning practices (gamification); information enrichment combining real and virtual elements (augmented reality); use of immersive experiences through visual and / or auditory simulations (virtual reality).

### Data Analysis Procedure

After debugging the database, data analysis includes the following statistical procedures:

- Checking the psychometric properties of the instrument so that it is valid and reliable. The reliability of the scale was analyzed through the calculation of Cronbach's Alpha and McDonald's Omega.
- The goodness indexes analyzed are: Chi-square ratio of degrees of freedom (χ^2^), Non-Standard Fit Index (NNFI), the Tucker-Lewis Index (TLI), the comparative adjustment index (CFI), the index of incremental adjustment (IFI), the root mean square error of approximation (RMSEA), composite reliability (CR), the Average Variance Extracted (AVE), and Maximum Shared Variance (MSV). As statistical software, SPSS V.26 and AMOS V.24 are used to check the modeling of structural equations (SEM) on the relationships between the items of the instrument.
- Verification of contrasts to verify compliance with the conditions necessary to perform parametric (t-Student) or non-parametric (Man-Whitney) tests on the self-perception variable in global digital competence.
- Application of the corresponding test to contrast in each range of teaching experience, if there are significant differences in the digital competence of teachers with respect to the question of using ICT resources in the classroom with their students (YES/NO), previously classified into three categories (resources ICT to consume, ICT resources to produce and emerging ICT resources).

## Analysis of the Results

### Psychometric Properties of the Instrument

**Fig. 1:**
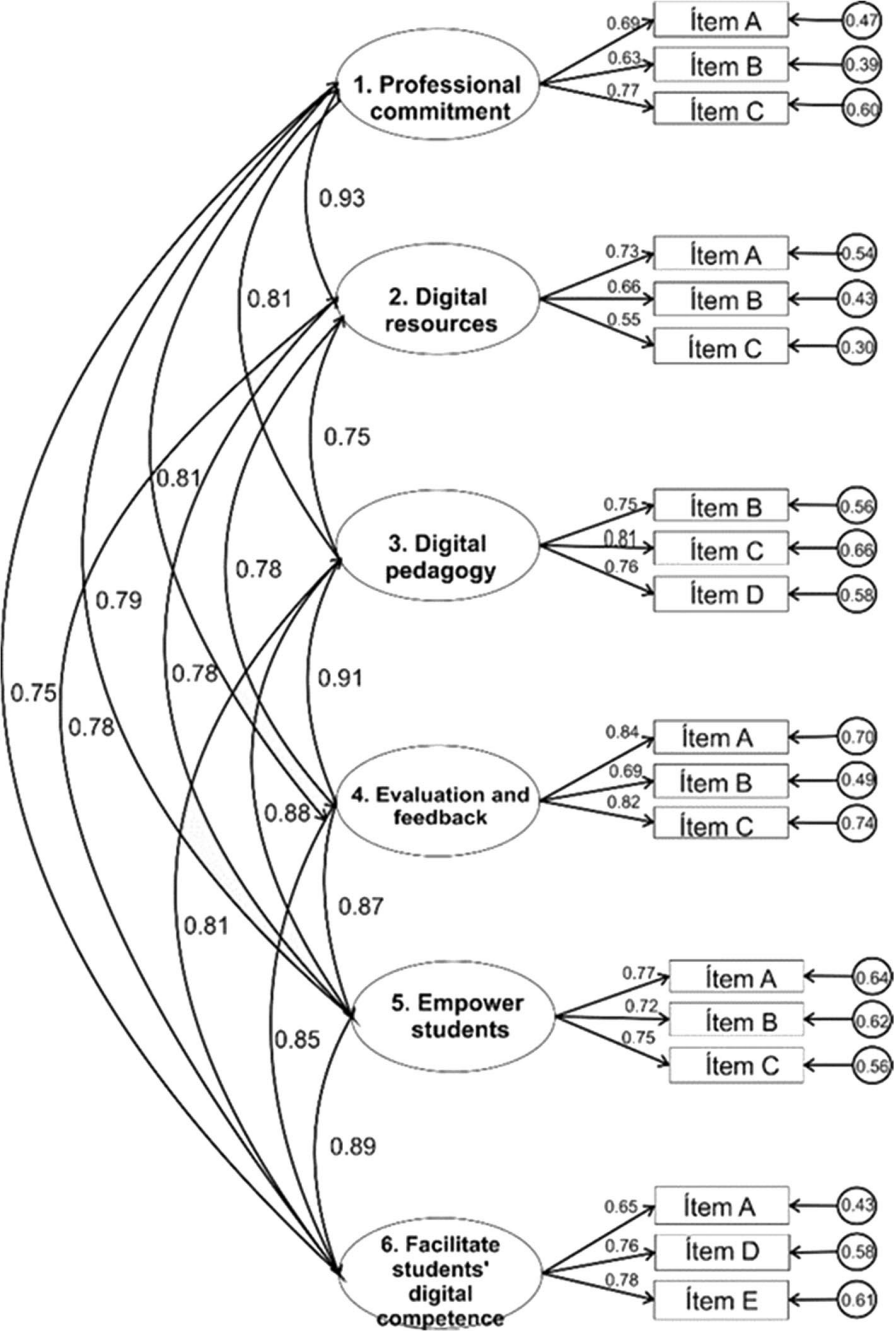
Structural diagram of the proposed model

In Thompson (2004) indications, the sample is divided into two subsamples to check the validity of the instrument. Bartlett’s test of sphericity (Bartlett’s test = 10,260.603; p < 0.001) and the Kaiser–Meyer–Olkin coefficient offers a value of 0.96, indicating the good fit of the model. The oblimin rotation by the maximum likelihood method detects six latent factors which explain 71.22% of the variance. In recommendations by Henson and Roberts (2006), items with weights less than 0.3 are eliminated since they correlate on different scales. The adjustment of the proposed model (Fig. 1) is quite acceptable, as indicated by the goodness indexes obtained from Table 2. The final version of the instrument with those items used in this study appears in Fig. 1.

**Table 2:** Confirmatory factor analysis of the Spanish version of DigCompEdu Check-In. Goodness of fit indexes of the factorial model

χ^2^	*p*	C.M	CFI	TLI	IFI	NNFI	RMR	RMSEA
4.97	0.001	25.45	0.96	0.94	0.955	0.94	0.032	0.06

Dimensions	Dim. 1	Dim. 2	Dim. 3	Dim. 4	Dim. 5	Dim. 6		
CR	0.74	0.71	0.82	0.83	0.79	0.78		
AVE	0.59	0.52	0.60	0.62	0.56	0.54		
MSV	0.87	0.87	0.82	0.82	0.79	0.79		

The internal consistency of Cronbach's Alpha and Mc Donal's Omega indicate very satisfactory levels of reliability, both in the dimensions of the instrument and in its overall set (Table 3).

**Table 3:** Item properties

Dimensions	Dim. 1	Dim. 2	Dim. 3	Dim. 4	Dim. 5	Dim. 6	TOTAL
Cronbach’s Alpha	0.73	0.65	0.77	0.80	0.78	0.78	0.93
McDonald’s Omega	0.67	0.69	0.73	0.78	0.75	0.75	0.99

On the other hand, it is verified through a descriptive study in which asymmetry and kurtosis have been taken into account that the data in the variable total digital competence is not normally distributed. Furthermore, the Kolmogorov–Smirnov test confirms this finding (KS = 0.057; gl = 2180; *p* < 0.05). Therefore, the Mann–Whitney test is used for subsequent contrasts.

### Differential Analysis between Digital Competence, use of Resources and Years of Teaching Experience

The following tables show the average of the total digital competence level of the teaching staff in each knowledge area, depending on whether or not they use the three types of ICT resources. Furthermore, the significance level and its effect size are shown in those cases where the comparative contrast between both responses is significant. Lenhard and Lenhard (2016) interpret the magnitude of the effect size according to Cohen (1988) and Hattie (1992). For Cohen, a value less than 0.4 is a small effect, between 0.5 and 0.7 a medium effect, and more than 0.8 a large effect. For Hattie (1992), the effect sizes refer to real educational contexts and, therefore, uses a more benign classification: values less than 0.1 with "developmental effects", between 0.2 and 0.3 with "teacher effects", and values greater than 0.4 with “Zone of desired effects”.

Table 4 shows the level of competence of the Arts and Humanities teachers. In teachers who have less than five years of teaching experience, there are hardly any significant differences in their level of digital competence regarding the use of ICT resources, except for creating videos, using augmented reality in didactic tasks and completing tests, this being the last one with the highest ES. Between 6 and 14 years of experience, not only do differences appear between those who complete tests or create videos, but also in the use of robotics, gamification and test creation, posters, concept maps, the latter being the one with the highest ES. However, it is the teachers who have the most teaching experience (over 15 years) where there are more significant differences between those who use ICT resources with their students compared to those who do not, practically in almost all the resources analyzed, being the creation of posters and concept maps which have the greatest ES.

**Table 4:** Digital competence in the use of ICT resources in the area of art and humanities

Years of experience	0–5 years				6–14 years				15 or more years			
	Yes	No	*p*	ES	Yes	No	*p*	ES	Yes	No	*p*	*ES*
Watch videos	39.59	32.00	–	–	43.48	37.00	–	–	39.07	34.00	–	–
See presentations	39.86	24.00	–	–	42.81	47.50	–	–	38.89	38.75	–	–
Complete test	42.02	28.58	*	0.88	47.17	36.65	*	0.85	42.79	30.96	*	0.96
See posters and concept maps	40.57	35.94	–	–	44.00	38.92	–	–	43.52	29.68	*	0.97
Create videos	47.44	36.39	*	0.75	52.31	37.24	*	0.50	46.97	34.08	*	1.03
Create web	38.00	39.43	–	–	46.20	42.54	–	–	51.74	35.22	*	1.14
Create test	40.67	39.00	–	–	52.78	39.53	*	1.04	51.69	36.35	*	0.81
Create posters and concept maps	42.73	37.37	–	–	50.41	36.40	*	1.17	48.85	33.65	*	1.21
Robotics	–	39.33	–	–	68,00	42,32	*	0.61	36.00	38.94	–	–
Gamification	41.94	35.42	–	–	47.63	37.14	*	0.78	44.79	37.15	*	0.43
Augmented reality	43.50	38.69	–	–	46.00	42,80	–	–	47.00	38.04	*	0.42
Virtual reality	46.75	38.19	*	0.73	47.17	4220	–	–	46.90	37.61	*	0.49

*Significance level at 0.05; *U* Mann–Whitney test; *ES* effect size

Table 5 shows the digital competence of teachers in the Science area. It can be seen how teachers with less than five years of experience are where the least significant differences appear in the level of digital competence based on the ICT resource used in the classroom (watch videos, see and create posters and concept maps, create websites and gamification). It is in the teaching staff with more than fifteen years of experience those that present the greatest significant differences in the level of digital competence, in the use of all the ICT resources analyzed, being the test compliance and creation of posters and conceptual maps those that have a greater. In teachers between the ages of 6 and 14, the use of the resources with the highest ES are also completing the test and creating digital posters and concept maps.

**Table 5:** Digital competence in the use of ICT resources in the area of sciences

Years of experience	0–5 years				6–14 years				15 or more years			
	Yes	No	*p*		Yes	No	*p*		Yes	No	*p*	
Watch videos	32.79	20.00	*	0.92	38.19	28.00	*	0.68	35.12	21.78	*	0.74
See presentations	31.45	–	–	–	37.78	27.00	–	–	33.92	25.88	*	0.25
Complete test	35.00	28.25	–	–	42.03	27.27	*	1.60	38.15	25.63	*	1.14
See posters and concept maps	33.50	27.00	*	0.84	41.68	33.48	*	0.68	40.85	29.25	*	0.95
Create videos	34.00	30.97	–	–	45.70	35.05	*	0.93	44.65	30.90	*	0.74
Create web	48.00	30.53	*	0.75	–	37.32	–	–	46.59	32.05	*	0.72
Create test	39.33	29.97	–	–	44.00	35.51	*	0.66	41.17	31.58	*	0.55
Create posters and concept maps	32.70	31.00	*	0.84	45.36	33.91	*	1.13	46.47	30.28	*	1.07
Robotics	–	31.45	–	–	53.00	36.62	*	0.66	68.50	33.07	*	0.45
Gamification	37.71	27.79	*	0.93	39.50	35.70	–	–	41.25	31.35	*	0.71
Augmented reality	31.50	31.44	–	–	43.50	36.05	–	–	52.00	32.87	*	0.43
Virtual reality	31.50	31.44	–	–	46.67	35.95	*	0.77	46.79	32.23	*	0.58

*Significance level at 0.05; *U* Mann–Whitney test, *ES* effect size

Table 6 shows the digital competence for teachers in the area of Health Sciences. It is observed that in the teachers with less than five years of experience, no significant differences appear in the use of emerging resources, only in the use of ICT resources to reproduce and produce, with the resources completing tests and seeing and creating posters and conceptual maps. that have a higher ES. In teachers between 6 and 14 years of experience, the resource that has the greatest difference in digital competence is the creation of concept maps, their ES being the largest of all. In teachers with more than 15 years of experience, differences appear in the use of all ICT resources, except for viewing multimedia presentations and robotics. The only ICT resources in which significant differences appear in the three groups of experience are completing tests, viewing posters and concept maps, and creating videos.

**Table 6:** Digital competence in the use of ICT resources in the area of health sciences

Years of experience	0–5 years				6–14 years				15 or more years			
	Yes	No	*p*		Yes	No	*p*		Yes	No	*p*	
Watch videos	37.67	19.83	*	0.90	39.68	32.00	–	–	34.95	21.20	*	0.53
See presentations	36.88	10.00	*	0.61	40.06	31.50	–	–	34.25	30.50	–	–
Complete test	40.55	25.55	*	1.26	41.89	31.75	*	0.66	39.06	26.06	*	1.12
See posters and concept maps	41.77	29.84	*	1.16	41.96	34.00	*	0.51	40.92	27.63	*	1.2
Create videos	41.85	30.12	*	1.01	45.10	35.18	*	0.84	41.80	30.17	*	0.80
Create web	36.00	36.08	–	–	53.40	37.27	*	0.93	54.00	32.43	*	0.83
Create test	40.46	33.29	*	0.78	42.00	37.75	–	–	43.76	31.84	*	0.72
Create posters and concept maps	43.07	31.05	*	1.13	45.46	32.97	–	1.03	42.98	31.20	*	0.74
Robotics	–	36.07	–	–	52.50	38.79	*	0.59	44.40	33.54	–	–
Gamification	38.46	32.75	–	–	43.87	35.95	*	0.68	39.58	31.99	*	0.48
Augmented reality	32.00	36.33	–	–	45.60	38.57	–	–	43.13	33.26	*	0.35
Virtual reality	39.33	35.75	–	–	43.83	38.69	–	–	46.20	32.61	*	0.53

*Significance level at 0.05; *U* Mann–Whitney test; *ES* Effect size

Table 7 shows the competence of the Engineering and Architecture faculty. In teachers with less than 5 years of experience, there is a significant difference in the level of competence between those who create videos, websites, posters and concept maps, complete tests, and use the gamification system. In the teachers with the greatest teaching experience, regardless of the range of years, significant differences appear in the use of all digital resources, except for robotics.

**Table 7:** Digital competence in the use of ICT resources in the area of engineering and architecture

Years of experience	0–5 years				6–14 years				15 or more years			
	Yes	No	*p*		Yes	No	*p*		Yes	No	*p*	
Watch videos	38.56	35.40	–	–	37.42	22.88	*	0.65	38.33	24.66	*	0.73
See presentations	39.26	24.33	–	–	36.18	–	–	–	36.89	11.50	*	0.39
Complete test	46.00	24.60	*	2.20	39.03	26.86	*	0.91	40.43	26.19	*	1.17
See posters and concept maps	41.43	31.15	–	–	42.48	31.10	*	1.04	43.40	29.03	*	1.35
Create videos	47.00	31.26	*	1.36	44.47	31.48	*	1.01	44.55	32.73	*	0.94
Create web	58.50	33.24	*	1.37	49.17	34.28	*	0.77	50.90	34.30	*	0.77
Create test	51.33	35.91	–	–	41.73	33.58	*	0.60	44.83	34.41	*	0.60
Create posters and concept maps	46.40	30.33	*	1.12	47.58	32.27	*	1.01	46.43	33.07	*	1.02
Robotics	58.00	37.15	–	–	36.13	36.19	–	–	40.10	36.27	–	–
Gamification	50.33	28.65	*	1.76	41.44	33.47	*	0.42	46.36	34.18	*	0.87
Augmented reality	43.50	37.59	–	–	46.00	35.01	*	0.41	46.70	34.61	*	0.62
Virtual reality	47.25	35.97	–	–	51.83	33.89	*	0.93	43.54	34.66	*	0.51

^*^Significance level at 0.05; *U* Mann–Whitney test, *ES* Effect size

Finally, Table 8 shows the teachers in the area of Social and Legal Sciences. Regardless of the range of teaching experience, significant differences appear in the level of digital competence in practically the use of almost all digital resources. It is the creation of websites, digital posters and concept maps where a higher ES appears, in both ranges of experience.

**Table 8:** Digital competence in the use of ICT resources in the area of social and legal sciences

Years of experience	0–5 years				6–14 years				15 or more years			
	Yes	No	*p*		Yes	No	*p*		Yes	No	*p*	
Watch videos	42.89	18.50	*	0.67	42.04	27.50	*	0.48	40.52	25.79	*	0.63
See presentations	41.91	–	–	–	41.62	26.50	*	0.29	39.67	32.33	–	–
Complete test	43.89	31.63	*	0.68	41.95	40.09	–	–	43.14	32.56	*	0.91
See posters and concept maps	45.52	31.77	*	1.01	43.17	34.40	*	0.66	44.02	31.93	*	1.06
Create videos	45.78	36.44	*	0.80	44.77	33.42	*	1.03	45.46	32.77	*	1.15
Create web	54.78	39.05	*	1.11	48.19	39.17	*	0.73	51.51	36.43	*	0.95
Create test	48.68	37.32	*	0.96	44.86	39.07	*	0.50	46.71	35.88	*	0.84
Create posters and concept maps	47.54	35.16	*	1.09	44.39	37.21	*	0.56	47.02	34.44	*	1.03
Robotics	55.90	40.34	*	0.82	44.00	41.24	–	–	55.16	38.14	*	0.68
Gamification	46.21	37.15	*	0.67	44.36	38.46	*	0.51	45.29	36.53	*	0.69
Augmented reality	52.56	39.86	*	0.76	46.45	40.36	*	0.38	53.88	37.17	*	0.85
Virtual reality	54.50	39.48	*	0.94	45.27	40.93	–	–	53.93	37.52	*	0.80

*Significance level at 0.05; *U* Mann–Whitney test, *ES* Effect size

## Discussions

The purpose of this study was: on the one hand, to know the level of digital competence of Higher Education teachers in the different areas of knowledge in relation to whether or not they use different digital courses in the classroom; and, on the other hand, to know if there are significant differences between the two types of teachers, all classified into three ranges of teaching experience (from 0 to 5 years, from 6 to 14 years, and more than 15 years).

In relation to those digital resources in which there are less significant differences between the teachers who use them and those who do not, we can observe that they are watching videos or multimedia presentations, in almost all age ranges and areas of knowledge, except in the area of art and humanities. Perhaps a possible explanation for these results is due to the statements of Maharaj-Sharma et al. (2017) who stated that the use of this type of resources is common for all teachers, although it offers the same benefits as if teachers use conventional presentations (Cosgun Ögeyik, 2017), perhaps caused by a democratization process with respect to the variables analyzed. Another resource in relation to previous research is robotics. Our results show that in all areas there are differences in the level of competence between both types of teaching staff, although not in all ranges of years of experience. These results are corroborated by Esteve-Mon et al. (2019) since they affirm that, to this day, there is still a lack of training in this type of resources, but contradictory to those of Román-Graván et al. (2020) as they stated that there were no differences in the use of this resource between different age ranges or study areas.

In relation to the years of experience, teachers with 15 or more years of experience are the group with the highest number of significant differences (teachers who use ICT resources versus those who do not), there being consensus in all areas of knowledge. These results on the negative relationship in the variable years of experience have been corroborated by other studies such as those of López Belmonte et al. (2020) and Oleksiuk and Oleksiuk (2020) which affirmed the younger teachers tend to implement more innovative methodologies through emerging. Following this reasoning, for an effective application of ICT, a significant dedication of time and effort is necessary, a factor for which many university professors do not develop these practices with technologies (Cabezas et al., 2020). The conception of this lack is justified not by the time that the teachers require to use them, but by the previous preparation of the lesson, “give me a year off to create an electronic program …. Sure! Then I will consider making more effort with the ICT in my classroom” (Prieto-Rodriguez, 2016, p. 6).

In relation to the differences in the teaching staff between those who use digital resources and those who do not, based on each area of knowledge, it is observed that in all the areas of knowledge there are significant differences regarding the level of digital competence when using ICT resources in teachers who have 15 or more years of experience. Specifically, it is the area of Sciences where significant differences appear in all the ICT resources analyzed, followed by the areas of Engineering and Social Sciences. Furthermore, these results reveal that the teachers who develop their competence are those who integrate ICT in the classroom. Together, these results can be taken as a reflection of the implementation of ICT that different authors have studied as those capable of enriching and improving the teaching–learning process (Miller & Nourbakhsh, 2016; Radianti et al., 2020). This development of specific digital competences, referring to the management and critical use of integrated technologies in the training context, constitutes one of the objectives of the Horizon 2019 report (Alexander et al., 2019). This is fundamental, since the uses of the innovative technologies and methodologies that derive from their implementation in the classroom turn out to be key tools for educational development due to the multiple benefits they produce in students (Parra-González et al., 2020; Sya & Helmanto, 2020).

For this reason, lifelong learning is a good resource to bring teachers closer to an inquiring and creative teaching model that enables a pedagogical practice aimed at the effective use of ICT resources (Barragán-Sánchez et al., 2020; Liesa-Orús et al., 2020). However, we consider that in addition to having sufficient, up-to-date and properly functioning computer resources, the key factor to opt for the possibility of applying technologies in educational contexts is time. Not only is it enough to have free time to carry out this permanent training, but from the university and government institutions they must modify and make the teaching loads of the teachers more flexible (Alexander et al., 2019).

## Conclusions and Future Works

Universities have progressively incorporated ICT into their classrooms, but it is true that a global strategy has not been addressed on what the training of students and teachers entails in terms of teaching digital competence. This fact seems to be glimpsed in the policies that the Ministries of Education begin to implement. However, the implementation of training environments for students and teachers in the acquisition of digital skills is not yet being implemented correctly. Therefore, it is relevant to study what are the handicaps that teachers present in this case and link them with theories and learning models on digital competences such as the TPACK model (Technological Pedagogy and Content Knowledge) (Cabero-Almenara, 2014), or the European Digital Competence Framework for Teachers, through “DigCompEdu”.

In this study, a comparison is carried out in different age ranges, of the level of digital competence of higher education professors from the universities of Andalusia, among those professors who use digital resources as “consumers in the teaching–learning process”, as “ prosumers” or “emerging resources”. This level was measured with the help of the DigCompEdu Check-in instrument in different areas of knowledge (Arts and Humanities, Sciences, Social Sciences, Engineering and Architecture).

The results of this quantitative study have shown a fact that has already become apparent from other previous investigations (Silva et al., 2019; Guillén-Gámez et al., 2020a). This reality is the link between university teaching staff and the need for ICT training and training plans that have been carried out from educational institutions. However, it can be considered a good initial starting point to establish a common framework of needs in digital teaching competencies. At the same time, how to approach such training from an integrated perspective in the curriculum. Also, the findings show that those teachers who are in the range of 15 or more years of experience represent the group with the most training deficiencies in the use of the three types of ICT resources, a fact that also, it is replicated in all areas of knowledge.

Through the results and conclusions obtained, this study presents a starting point towards the recommendation to Andalusian university institutions on the need to design, plan, structure and implement training action. In this line, the acquisition of digital skills by higher education teachers at different levels and integrated within the projects and programs of the different subjects. All of this is presented as challenges that need further study through studies and research projects for the future, especially in the current times, where the development of digital skills by teachers; regardless of the educational level we are talking about, it is presented as essential for any teaching activity that wants to be considered quality.

Likewise, the theoretical assumptions proposed by other studies on the same subject are empirically corroborated. In this sense, referring to the need to have scientifically validated competence assessment tools, the low-medium competence level of university teachers and the importance of establishing personalized training plans (Rodríguez-Hoyos et al., 2021). All this, under the same Digital Competence Framework, as DigCompEdu. In this last sense, the TPACK Model, from which DigCompEdu emanates, corroborates the above: necessary training in technological, pedagogical and content content (Mutiani et al., 2021). Along these lines, the study by Hidson (2021) suggests that collaborative pedagogy can be a fundamental variable for the development of digital skills.

Regarding the limitations of this study, it would be necessary to critically reflect on what its weaknesses are and how to improve them through future work. Perhaps the main weakness is the design of the sample. For this study an intentional sampling was used, which indicates that the sample is not random and, therefore, the results obtained cannot be extrapolated to the general population of all areas of knowledge of the teaching staff. This weakness could be improved in future studies through probabilistic samples, or on the contrary, if it were not possible, with intentional samples that make it possible to collect data not only from one country or continent, but from several (Europe, North America, South America, Africa…). The purpose of this is to expand the triangulation of factors analyzed in the digital competence of teachers. In this way, it would be possible to expand the size of the sample, making it more representative of the entire Higher Education community.

Another limitation of the study is the type of variables included. We have found that in some digital resources, some areas of knowledge and years of experience the level of competence varies. Probably, there are other variables that can also influence the biological sex of the individual, which has been deeply studied previously in the literature, but perhaps it could be taken a step further, classifying it not only as male or female, but also the gender identity. Another variable to take into account is the use of technology at home, that is, what type of devices teachers use at home for work, leisure or personal enjoyment, which can have an educational impact on said competence. Finally, these limitations could be analyzed in a more real context, through training courses with pre and posttest designs. In this way, it would be possible to contextualize the weaknesses of teachers, and train and specialize teachers in those types of resources that need it most, adapted to their area of knowledge, years of experience, or other variables.

These much-needed training courses for teachers to improve their proficiency levels could begin through courses MOOC that allow educators at all educational levels—although more focused on higher education levels—to automatically self-assess their skills in teaching, fast and scalable way, instead of accessing commonly received training, and that allows covering the difficulties that each teacher may encounter when it comes to self-diagnosing their digital skills and training in them appropriately. The self-assessment will therefore provide you with an answer to questions such as, at what level of digital competence am I currently? What content and training practices are necessary to meet my needs? sufficient digital competence for my teaching practice? The research and development of all these aspects provides an interesting framework for action on training action programs whose implementation goes beyond addressing in a limited and specific way the way in which the training needs of university teachers have been covered, and not university students.

## References

[CR1] Alexander, B., Ashford-Rowe, K., Barajas-Murph, N., Dobbin, G., Knott, J., McCormack, M., & Weber, N. (2019). *Horizon Report 2019 Higher Education Edition*. EDUCAUSE.

[CR2] Auma OM, Achieng OJ (2020). Perception of teachers on effectiveness of online learning in the wake of COVID-19 pandemic. IOSR Journal of Humanities and Social Science (IOSR-JHSS).

[CR3] Barragán-Sánchez R, Corujo-Vélez M-C, Palacios-Rodríguez A, Román-Graván P (2020). Teaching digital competence and eco-responsible use of technologies: Development and validation of a scale. Sustainability.

[CR4] Cabero-Almenara, J. (2007). *Posibilidades de la teleformación en el Espacio Europeo de Educación Superior*. Octaedro.

[CR5] Cabero-Almenara, J. (2014). *La formación del profesorado en TIC: Modelo TPACK*. Secretariado de Recursos Audiovisuales y Nuevas Tecnologías de la Universidad de Sevilla.

[CR6] Cabero-Almenara J, Barroso-Osuna J, Gutiérrez-Castillo JJ, Palacios-Rodríguez A (2020). Validación del cuestionario de competencia digital para futuros maestros mediante ecuaciones estructurales. Bordón Revista De Pedagogía.

[CR7] Cabero-Almenara J, Estrada-Vidal L, Gutiérrez-Castillo JJ (2017). Diseño y validación de un instrumento de evaluación de la competencia digital del estudiante universitario. Revista Espacios.

[CR8] Cabero-Almenara, J., & Llorente-Cejudo, C. (2005). Las plataformas virtuales en el ámbito de la teleformación. *Revista electrónica Alternativas de Educación y Comunicación,* 1–24.

[CR9] Cabero-Almenara J, Palacios-Rodríguez A (2020). Marco Europeo de Competencia Digital Docente «DigCompEdu» y cuestionario «DigCompEdu Check-In». EDMETIC Revista de Educación Mediática y TIC.

[CR10] Cabezas AR, Domínguez MCM, Navío EP, Rivilla AMM (2020). Formación del Profesorado Universitario en la Competencia Digital. Píxel-Bit. Revista de Medios y Educación.

[CR11] Cacheiro, M. L., Sánchez, C., & González, J. M. (2019). The digital Competence of the Social Educators as a User and Creator of Educational Resources. In *Online, Open and Flexible Higher Education Conference (OOFHE), EADTU*.

[CR12] Calvani A, Cartelli A, Fini A, Ranieri M (2008). Models and instruments for assessing digital competence at school. Journal of E-learning and Knowledge Society.

[CR13] Castro E, Cecchi F, Salvini P, Valente M, Buselli E, Menichetti L, Dario P (2018). Design and impact of a teacher training course, and attitude change concerning educational robotics. International Journal of Social Robotics.

[CR14] Cejas R, Navío A, Barroso-Osuna J (2016). Las competencias del profesorado *universitario desde el Modelo TPACK (conocimiento tecnológico y pedagógico del contenido). Revista Pixelbit*. Revista de Medios y Educación.

[CR15] Cohen J (1988). The effect size index: D. Statistical Power Analysis for the Behavioral Sciences.

[CR16] Cosgun Ögeyik M (2017). The effectiveness of Power point presentation and conventional lecture on pedagogical content knowledge attainment. Innovations in Education and Teaching International.

[CR17] Daniel SJ (2020). Education and the COVID-19 pandemic. Prospects.

[CR18] Drijvers P, Doorman M, Boon P, Reed H, Gravemeijer K (2010). The teacher and the tool: Instrumental orchestrations in the technology-rich mathematics classroom. Educational Studies in mathematics.

[CR19] Esteve-Mon FM, Adell-Segura J, Nebot MÁL, Novella GV, Aparicio JP (2019). The development of computational thinking in student teachers through an intervention with educational robotics. Journal of Information Technology Education: Innovations in Practice.

[CR20] Farjon D, Smits A, Voogt J (2019). Technology integration of pre-service teachers explained by attitudes and beliefs, competency, access, and experience. Computers & Education.

[CR21] Finnegan M, Ginty C (2019). Moodle and social constructivism: is moodle being used as constructed? A case study analysis of moodle use in teaching and learning in an irish higher educational institute. All Ireland Journal of Higher Education.

[CR22] García-Esteban S (2017). Do video learning objects develop digital competence in teacher training ?. RAEL: revista electrónica de lingüística aplicada.

[CR23] Ghomi, M., & Redecker, C. (2018). *Digital Competence of Educators (DigCompEdu): Development and Evaluation of a Self-Assessment Instrument for Teachers’ Digital Competence*. Joint Research Center. http://www.doi.org/10.5220/0007679005410548

[CR24] Gómez-García M, Trujillo-Torres JM, Aznar-Díaz I, Cáceres-Reche MP (2018). Augment reality and virtual reality for the improvement of spatial competences in Physical Education. Journal of Human Sport and Exercise.

[CR25] González-Martínez J, Esteve-Mon FM, Larraz Rada V, Espuny Vidal C, Gisbert Cervera M (2018). INCOTIC 2.0: una nueva herramienta para la autoevaluación de la competencia digital del alumnado universitarioINCOTIC 2.0: A new self-assessment tool for digital competences at the university studies. Profesorado: revista de currículum y formación del profesorado.

[CR26] Guillén-Gámez FD, Mayorga-Fernández MJ (2020). Quantitative-comparative research on digital competence in students, graduates and professors of faculty education: An analysis with ANOVA. Education and Information Technologies.

[CR27] Guillén-Gámez FD, Mayorga-Fernández MJ, Álvarez-García FJ (2018). A study on the actual use of digital competence in the practicum of education degree. Technology, Knowledge and Learning.

[CR28] Guillén-Gámez FD, Mayorga-Fernández MJ, Bravo-Agapito J, Escribano-Ortiz D (2020). Analysis of teachers’ pedagogical digital competence: Identification of factors predicting their acquisition. Technology, Knowledge and Learning.

[CR29] Guillen-Gamez FD, Mayorga-Fernández MJ, Del Moral MT (2020). Comparative research in the digital competence of the pre-service education teacher: face-to-face vs blended education and gender. Journal Of E-Learning And Knowledge Society.

[CR30] Guillén-Gámez FD, Peña MP (2020). Análisis Univariante de la Competencia Digital en Educación Física: un estudio empírico. Retos: nuevas tendencias en educación física deporte y recreación.

[CR31] Hattie J (1992). Measuring the effects of schooling. Australian Journal of Education.

[CR32] Henson RK, Roberts JK (2006). Use of exploratory factor analysis in published research: Common errors and some comment on improved practice. Educational and Psychological Measurement.

[CR33] Hernandez-Pozas O, Carreon-Flores H (2019). Teaching international business using virtual reality. Journal of Teaching in International Business.

[CR34] Hidson E (2021). Pedagogy by proxy: teachers’ digital competence with crowd-sourced lesson resources. Pixel-Bit. Revista de Medios y Educación.

[CR35] INTEF (2017). Marco Común de Competencia Digital Docente. *Instituto Nacional de Tecnologías Educativas y de Formación del Profesorado*.

[CR36] Jackson EA (2017). Impact of MOODLE platform on the pedagogy of students and staff: Cross- curricular comparison. Education and Information Technologies.

[CR37] Kyaw BM, Saxena N, Posadzki P, Vseteckova J, Nikolaou CK, George PP, Car LT (2019). Virtual reality for health professions education: Systematic review and meta-analysis by the digital health education collaboration. Journal of Medical Internet Research.

[CR38] Leiva Núñez J, Ugalde Meza L, Llorente-Cejudo C (2018). El modelo tpack en la formación inicial de profesores: modelo Universidad de Playa Ancha (UPLA), Chile. Pixel-Bit. Revista de Medios y Educación.

[CR39] Lenhard W, Lenhard A (2016). Calculation of Effect Sizes. Psychometrica.

[CR40] Liesa-Orús M, Latorre-Cosculluela C, Vázquez-Toledo S, Sierra-Sánchez V (2020). The technological challenge facing higher education professors: perceptions of ICT tools for developing 21st century skills. Sustainability.

[CR41] López Belmonte J, Pozo Sánchez S, Fuentes Cabrera A, Romero Rodríguez JM (2020). Uses and integration of augmented reality in the educational cooperatives of Andalusia (Spain). JOTSE: Journal of Technology and Science Education.

[CR42] Maharaj-Sharma R, Sharma A (2017). Using ICT in secondary school science teaching-what students and teachers in trinidad and tobago say?. European Journal of Education Studies.

[CR43] Maharaj-Sharma R, Sharma A, Sharma A (2017). Using ICT-based instructional technologies to teach science: Perspectives from teachers in Trinidad and Tobago. Australian Journal of Teacher Education.

[CR44] MCIU. (2019). *Datos y cifras del sistema universitario español. Publicación 2018–2019*. Ministerio de Ciencia, Innovación y Universidades.

[CR45] Miller, D. P., & Nourbakhsh, I. (2016). Robotics for education. In *Springer handbook of robotics* (pp. 2115–2134). Springer, Cham. 10.1007/978-3-319-32552-1_79

[CR46] Miravete ÁDF (2018). La competencia digital del alumnado de Educación Secundaria en el marco de un proyecto educativo TIC (1: 1). Edutec. Revista Electrónica de Tecnología Educativa.

[CR47] Murphy MPA (2020). COVID-19 and emergency eLearning: Consequences of the securitization of higher education for post-pandemic pedagogy. Contemporary Security Policy.

[CR48] Mutiani M, Supriatna N, Abbas EW, Rini TPW, Subiyakto B (2021). Technological, pedagogical, content knowledge (TPACK): A discursions in learning innovation on social studies. The Innovation of Social Studies Journal.

[CR49] Oleksiuk, V., & Oleksiuk, O. (2020). Exploring the potential of augmented reality for teaching school computer science. *3rd International Workshop on Augmented Reality in Education, 2731*, 91–107.

[CR50] Ortiz PA (2020). Teaching in the time of COVID-19. Biochemistry and Molecular Biology Education.

[CR51] Padilla-Hernández AL, Gámiz-Sánchez VM, Romero-López MA (2020). Evolución de la competencia digital docente del profesorado universitario: Incidentes críticos a partir de relatos de vida. Educar.

[CR52] Parra-González ME, López Belmonte J, Segura-Robles A, Fuentes Cabrera A (2020). Active and emerging methodologies for ubiquitous education: Potentials of flipped learning and gamification. Sustainability.

[CR53] Prieto-Rodriguez E (2016). It just takes so much time!: A study of teachers' use of ICT to convey relevance of mathematical content. International Journal for Technology in Mathematics Education.

[CR54] Radianti J, Majchrzak TA, Fromm J, Wohlgenannt I (2020). A systematic review of immersive virtual reality applications for higher education: Design elements, lessons learned, and research agenda. Computers & Education.

[CR55] Redecker C, Punie Y (2017). Digital Competence of Educators DigCompEdu.

[CR56] Rodríguez-Hoyos C, Fueyo Gutiérrez A, Hevia Artime I (2021). Competencias digitales del profesorado para innovar en la docencia universitaria. Analizando el uso de los dispositivos móviles. Píxel-Bit. Revista De Medios Y Educación.

[CR57] Román-Graván P, Hervás-Gómez C, Martín-Padilla AH, Fernández-Márquez E (2020). Perceptions about the use of educational robotics in the initial training of future teachers: A study on steam sustainability among female teachers. Sustainability.

[CR58] Romero-Tena R, Barragán-Sánchez R, Llorente-Cejudo C, Palacios-Rodríguez A (2020). The challenge of initial training for early childhood teachers. A cross sectional study of their digital competences. Sustainability.

[CR59] Ruiz ABM, Sánchez FAG, Pina FH (2015). Cuestionario para el estudio de la actitud, el conocimiento y el uso de TIC (ACUTIC) en Educación Superior. Estudio de fiabilidad y validez. Revista Interuniversitaria De Formación Del Profesorado.

[CR60] Santos Espino JM, Afonso Suárez MD, González-Henríquez JJ (2020). Video for teaching: Classroom use, instructor self-production and teachers’ preferences in presentation format. Technology, Pedagogy and Education.

[CR61] Siddiq F, Scherer R, Tondeur J (2016). Teachers’ emphasis on developing students’ digital information and communication skills (TEDDICS): A new construct in 21st century education. Computers & Education.

[CR62] Silva JS, Usart MU, Lázaro-Cantabrana JLLC, Silva J, Usart M, Lázaro-Cantabrana JL (2019). Teacher’s digital competence among final year Pedagogy students in Chile and Uruguay. Comunicar. Media Education Research Journal.

[CR63] Sya MF, Helmanto F (2020). Writing poster at higher education: victor schwab theory analysis. Wanastra: Jurnal Bahasa dan Sastra.

[CR64] Thompson B (2004). Exploratory and confirmatory factor analysis: Understanding concepts and applications.

[CR65] Tondeur J, Aesaert K, Prestridge S, Consuegra E (2018). A multilevel analysis of what matters in the training of pre-service teacher’s ICT competencies. Computers & Education.

[CR66] Vázquez-Cano E, Marín-Díaz V, Oyarvide WRV, Meneses EL (2020). Use of augmented reality to improve specific and transversal competencies in students. Int J Learn Teach Educ Res.

